# Interleukin 1 level, cognitive performance, and severity of depressive symptoms in patients treated with systemic anticancer therapy: a prospective study

**DOI:** 10.3325/cmj.2019.60.166

**Published:** 2019-04

**Authors:** Justyna Jasionowska, Monika Talarowska, Ewa Kalinka, Aleksandra Skiba, Janusz Szemraj, Iwona Mikołajczyk, Piotr Gałecki

**Affiliations:** 1 Department of Adult Psychiatry, Medical University of Lodz, Lodz, Poland; 2 Department of Oncological Surgery and Breast Diseases, Polish Mother’s Memorial Hospital Research Institute, Lodz, Poland; 3 Department of Medical Biochemistry, Medical University of Lodz, Lodz, Poland; 4 Sub-unit of Diagnostics and Oncological Therapy, Department of Chemotherapy, Regional Oncological Centre, Voivodeship Specialist Hospital, Lodz, Poland; * The first two authors contributed equally.

## Abstract

**Aim:**

To assess the relationship between cognitive functions, severity of depressive symptoms, and expression of interleukin 1 (IL)-1 in patients treated with systemic anticancer therapy.

**Methods:**

This prospective study, conducted in 2017-2018, involved 55 patients (56% men) subjected to systemic anticancer therapy. Forty-one patients had lung cancer (74.55%) and 14 had breast cancer (25.45%). Patients’ mean age was 55.5 ± 9.3 (from 26 to 65 years). Neuropsychological tests were conducted twice: on the day of qualifying for the study before the start of chemotherapy and after the end of the full treatment cycle. We assessed patients’ cognitive functioning using Trail Making Test A&B (TMT), Stroop Color-Word Interference Test, and Verbal Fluency Test (VFT). Severity of depressive symptoms and the level of IL-1 expression were also examined.

**Results:**

After chemotherapy, patients had significantly lower expression of IL-1α (*P* < 0.005) and IL-1β (*P* < 0.001) at the protein level. They also had lower severity of depressive symptoms (borderline significant, *P* = 0.063), needed more time to complete the first part of the Stroop test (*P* = 0.03), and had worse score on the first part of the VFT (*P* < 0.001). Before chemotherapy there was a significant negative correlation between IL-1β expression and the speed at which the first part of the TMT test was completed.

**Conclusions:**

The severity of depressive symptoms after chemotherapy was lower than before chemotherapy. Patients’ cognitive performance did not significantly deteriorate after chemotherapy, except the performance at the first part of the Stroop test and the first part of the VFT.

The cancer incidence in Europe is particularly high – about 20% of all cancer deaths and almost 25% of cases of cancer worldwide are recorded in Europe (1). In this sense, Poland is no exception; in 2015, malignant cancer was the second most common cause of death among Polish citizens (about 27% of all deaths in men and 24% in women) (2). Cancer is also the most common cause of premature mortality, particularly among women, accounting for as much as 33% of deaths among young women and about 50% of deaths among middle-aged women. According to the Polish National Cancer Registry, there were slightly more than 159 200 new cases and about 95 600 cancer deaths in 2014 (3).

One of the side effects of systemic anticancer therapy are changes in cognitive function, referred to as chemobrain, chemofog, or chemotherapy-related cognitive impairment (4,5). This phenomenon is characterized by impaired visual and verbal memory, working memory, concentration, language skills, response time, and motor skills (5,6). Chemobrain affects from 17% to 75% of all cancer patients subjected to therapy (6) and is usually mild and transient, with escalation during anticancer treatment.

Although most studies indicate that chemobrain symptoms are short-term, it was reported that in 35% of patients with cancer remission they persisted for several years (7). The etiology of chemobrain is complex and non-uniform, with influencing factors ranging from those related to the presence of cancer and its treatment process to patient-related ones (6). The causes of cognitive disorders after chemotherapy include changes in central nervous system metabolism, damage to neurons, decrease in neurotransmitters activity, impact of procedures such as anesthesia, use of additional pharmacological agents, coexisting diseases, as well as changes in the release of proinflammatory cytokines, which have recently been of particular interest among researchers (6,8). Cytostatic drugs are believed to marginally cross the blood-brain barrier, but their neurotoxic effects are probably related to the action of proinflammatory cytokines (9).

The mechanism of cognitive changes induced by cytokine action in the central nervous system is not fully understood. There are studies describing a sudden increase in the level of cytokines during the day after chemotherapy (10). Cognitive impairment is often described as an element of the so-called sickness behavior characterized by an increase in cytokines such as interleukin 1 (IL-1), interleukin 2 (IL-2), or tumor necrosis factor alpha (TNF-α). This phenomenon is one of the etiological factors of depressive disorders (6). In the hippocampus and other regions rich in receptors for cytokines, these protein mediators can induce inflammation through the oxidative mechanism and nitrosative mechanism (9). A relationship between a slightly intensified inflammatory state and a decrease in the hippocampus volume, which deteriorates memory functions, was also described (11,12). Changes in the hippocampus structure in the dominant hemisphere reduce the ability to learn and store information in memory, as well as the ability to recognize verbal material presented in a visual or auditory form (12).

In order to better understand the influence of cytokines, namely IL-1, on changes in cognitive processes associated with anticancer chemotherapy, we assessed the impact of chemotherapy-induced inflammatory reaction on cognitive functions, as well as on the severity of depressive symptoms. According to our knowledge, this is the first study on this issue, analyzing all three variables: level of interleukins, severity of depression, and cognitive performance among patients with cancer.

## Material and methods

### Patients

The study, conducted in 2017-2018, included patients hospitalized at the Department of Chemotherapy with the Sub-unit of Diagnostics and Oncological Therapy of the Regional Oncological Centre at the Voivodeship Specialist Hospital and the Department of Oncological Surgery of the Polish Mother’s Memorial Hospital Research Institute in Lodz.

The study started on March 1, 2017 (FPFV date) and ended on October 31, 2018 (LPLV date). Patients hospitalized in the mentioned wards were included in the study if they met the inclusion and exclusion criteria and agreed to participate in the study. Sixty-three people were included in the first stage of the study (60.32% men). Eight patients left the study without giving a reason (5 with lung cancer and 3 with breast cancer). In this group, only the first stage of the study was performed. Obtained results were not included in statistical analyzes.

The inclusion criteria were age between 25 and 65 years and the diagnosis of lung cancer or breast cancer not earlier than one month before the enrollment in the study. The exclusion criteria were metastases to the central nervous system, completed palliative and elective radiotherapy of the central nervous system, history of diagnosed axis I or II mental disorders (before commencement of cancer management), central nervous system traumas, inflammatory or autoimmune disorders, the level of intellectual functioning below average, excessive use of or addiction to psychoactive substances, pharmacotherapy that may negatively affect cognitive performance (besides chemotherapy), and unwillingness to give informed consent. Patients’ eligibility for the study was assessed by the same person – an oncologist. Medical data on the course of the disease were obtained directly from the patients, from attending physicians, and from medical records (with patients’ consent). Ultimately, those who agreed to participate in the study and underwent the entire treatment cycle were qualified to participate in the study (N = 55, 56% men). Each patient gave a written consent to participate in the study, and the study was approved by the Bioethics Committee of the Medical University of Lodz (No. RNN/497/13/KB).

The authors were not involved in the process of diagnosis or treatment at any stage of the study. Forty-one patients had lung cancer (74.55%) and 14 had breast cancer (25.45%). Patients’ mean age was 55.5 ± 9.3 years and ranged from 26 to 65 years.

Pharmacotherapeutic agents used were cycle-specific agents that destroy mainly cells in the cellular cycle, but provide lower effectiveness in relation to the G0 phase (cisplatin, carboplatin) and phase-specific agents that destroy cells in a particular cycle phase (antimetabolites: gemcitabine, pemetrexed; alkaloids: vinorelbine; taxoids: paclitaxel, docetaxel).

## Methods

### Cognitive function evaluation

The patients underwent the neuropsychological tests twice: on the day of qualifying for the study before the start of chemotherapy and after the end of the full treatment cycle. Cognitive performance was assessed by the same person (clinical psychologist, neuropsychologist). The average time between the first and the second stage of the study was 18 weeks. The second stage of the study took place within a week of taking the last dose of the drug. Only patients who completed the entire treatment cycle were included in the statistical analysis.

We analyzed the performance of information processing speed (Trail Making Test A & B, TMT), working memory and executive functions (TMT, the Stroop Color-Word Interference Test, verbal fluency test, VFT), and verbal fluency (VFT) (13). We wanted to ensure that participation in the study was not an additional burden for the patients. We created a parallel version of VFT for the assessment after chemotherapy because the participants were already familiar we the first version (the first version of the VFT included animals, words beginning with the letter “k” and “s”; the second version of the VFT included plants, words beginning with the letter “m” and “p”).

### Assessment of severity of recurrent depressive disorder symptoms

To evaluate the dynamics of severity of recurrent depressive disorder symptoms both before and after chemotherapy we used Hamilton Depression Rating Scale (HDRS, HAM-D) (14). We also used the CIDI questionnaire (version 3.0) (15), to assess the patient’s eligibility for the study.

### Determination of IL-1α and IL-1β expression at the mRNA and protein level

Ten mL of venous blood (two 5 mL test tubes) were collected by qualified medical personnel using sterile and disposable equipment, both before and after the chemotherapy. The procedure of IL-1α and IL-1β expression level assessment was described in detail in a previous study (16).

### Statistical analysis

Normality of distribution was tested with the Shapiro–Wilk test. The groups were compared using the Wilcoxon matched pairs test with the Bonferroni correction in each case. The correlations between the analyzed variables were evaluated using Spearman’s rank correlation coefficient. The level of statistical significance was set at *P* < 0.05 (17). The statistical analysis was performed with STATISTICA PL, version 13.1 (StatSoft Polska Sp., Krakow, Poland).

## Results

Patients before chemotherapy had somewhat greater severity of depressive symptoms than after chemotherapy, ie, the difference was borderline significant (*P* = 0.06) (Table 1). The mean severity of depressive symptoms before chemotherapy met the criteria of a mild depressive episode, while the mean the severity after chemotherapy did not reach the level of diagnostic significance. The expression of IL-1α (*Z* = 4.63, *P* < 0.005) and IL-1β (*Z* = 5.32, *P* < 0.001) at the protein level significantly decreased after chemotherapy (Table 1).

**Table 1:** The severity of depressive symptoms and expression of interleukin (IL)-1α and IL-1β in patients with cancer before and after systemic anticancer therapy

Variable	Before			After			Z*	P*
median (IQR)	minimum	maximum	median (IQR)	minimum	maximum
Hamilton Depression Rating Scale	8 (5-13)	1.00	19.00	5 (3-7)	0.00	26.00	1.857	0.063
IL-1α mRNA (2^-ΔΔct^)	0.177 (0.142-0.21)	0.11	0.28	-	-	-	-	-
IL-1α protein (pg/mL)	8.841 (6.790-10.36)	5.54	14.19	7.431 (6.661-9.871)	4.69	12.23	4.631	<0.005
IL-1β mRNA (2^-ΔΔct^)	0.039 (0.036- 0.05)	0.02	0.11	-	-	-	-	-
IL-1β protein (pg/mL)	1.981 (1.741-2.19)	1.19	3.13	1.815 (1.631-2.151)	1.28	2.75	5.316	<0.001

*the Wilcoxon test.

### Assessment of cognitive performance

No differences were found in the performance on TMT test before and after chemotherapy. For the Stroop Color-Word Interference Test and the Verbal Fluency Test, the differences were significant only for the first part of the tests. Patients after chemotherapy needed significantly more time to complete the first part of the Stroop test (RCNb-reading color name in black) (*P* = 0.03). They also had a significantly worse score on the first part of the Verbal Fluency Test (semantic category) (*P* < 0.001) (Table 2).

**Table 2:** Cognitive performance in patients with cancer before and after systemic anticancer therapy*

Variable	Before			After			Z^†^	*P^†^*
	median (IQR)	minimum	maximum	median (IQR)	minimum	maximum		
Stroop Test RCNb time (seconds)	30 (25-37)	21.00	59.00	30.51 (26-38)	22.00	135.00	2.228	0.03
Stroop Test NCWd time (seconds)	70 (55-84)	41.00	112.00	56.51 (51.51-72.51)	38.00	172.00	1.591	0.11
Fluency part a	18 (14-22)	8.00	34.00	11 (10-18)	3.00	24.00	4.287	<0.001
Fluency part b	9 (7-13)	4.00	16.00	10.51 (8-13)	4.00	18.00	0.326	0.69
Fluency part c	13 (10-17)	3.00	25.00	11 (8.5-16.5)	1.00	21.00	0.417	0.68
TMT part A time (seconds)	36 (25-55)	16.00	100.00	36 (22-44)	13.00	104.00	0.524	0.76
TMT part B time (seconds)	90 (69-128)	44.00	245.00	84 (60-112)	36.00	174.00	1.591	0.11

*RCNb – reading color names in black; NCWd – naming color of word – different; TMT – Trail Making Test; before – on the day of qualifying for the study before the start of chemotherapy; after – after the end of the full treatment cycle.

†the Wilcoxon test.

### Correlations

Before chemotherapy, there were some weak and non-significant correlations – between increased IL-1α expression at the mRNA and protein levels and both severity of depressive symptoms and cognitive performance impairment in each of the tests conducted and between increased IL-1 β expression at the mRNA and protein levels and lower depressive symptoms and better cognitive performance on the third part of VFT (part c). A significant negative correlation was observed between IL-1β expression and the speed at which the first part of the TMT test was performed (Table 3).

**Table 3:** Correlations between IL-1α and IL-1β levels, Hamilton Depression Rating Scale (HDRS) score, and cognitive performance in patients with cancer before systemic anticancer therapy*

Variable	IL-1α mRNA (2^-ΔΔct^)		IL-1α protein (pg/mL)		IL-1β mRNA (2^-ΔΔct^)		IL-1β protein (pg/mL)	
	Spearman’s rho	*P*	Spearman’s rho	*P*	Spearman’s rho	*P*	Spearman’s rho	*P*
HDRS	0.046	0.760	0.004	0.977	-0.193	0.195	-0.222	0.133
Stroop Test RCNb time (seconds)	-0.063	0.675	-0.053	0.722	0.062	0.677	0.082	0.583
Stroop Test NCWd time (seconds)	-0.166	0.265	-0.128	0.390	0.172	0.248	0.030	0.842
Fluency part a	-0.178	0.231	-0.183	0.218	-0.241	0.103	-0.244	0.098
Fluency part b	-0.102	0.494	-0.097	0.516	-0.191	0.198	-0.116	0.436
Fluency part c	-0.021	0.888	-0.020	0.896	0.115	0.441	0.227	0.126
TMT part A time (seconds)	0.062	0.684	0.067	0.657	-0.113	0.453	-0.310	0.036
TMT part B time (seconds)	0.047	0.754	0.032	0.832	-0.005	0.971	-0.161	0.285

*HDRS – Hamilton Depression Rating Scale; RCNb – reading color names in black; NCWd – naming color of the word – different; TMT – Trail Making Test.

After chemotherapy, an increased IL-1α expression at the mRNA and protein levels weakly and non-significantly correlated with both severity of depressive symptoms and cognitive performance impairment in each of the tests conducted. In the case of IL-1β, there was no significant correlation for any of the analyzed variables (Table 4).

**Table 4:** Correlations between IL-1α and IL-1β levels, Hamilton Depression Rating Scale (HDRS) score, and cognitive performance in patients with cancer after systemic anticancer therapy*

Variable	IL-1α protein (pg/mL)		IL-1β protein (pg/mL)	
	Spearman’s rho	*P*	Spearman’s rho	*P*
HDRS	0.055	0.740	-0.234	0.152
Stroop Test RCNb time (seconds)	-0.007	0.969	0.090	0.586
Stroop Test NCWd time (seconds)	-0.034	0.836	0.135	0.411
Fluency part a	-0.181	0.270	-0.290	0.073
Fluency part b	-0.047	0.777	-0.072	0.662
Fluency part c	-0.008	0.961	0.216	0.187
TMT part A time (seconds)	0.145	0.385	-0.239	0.148
TMT part B time (seconds)	0.004	0.982	0.091	0.588

*HDRS – Hamilton Depression Rating Scale; RCNb – reading color names in black; NCWd – naming color of word – different; TMT – Trail Making Test.

## Discussion

We did not find any significant deterioration in the patients' cognitive performance after the chemotherapy cycle. However, the severity of depressive symptoms decreased. Both before and after chemotherapy there was a non-significant relationship between the IL-1α expression at the mRNA and protein level with the severity of depressive symptoms and cognitive performance impairment in each of the tests conducted.

The studies conducted so far present a wide range of results concerning the severity of depressive symptoms in cancer patients undergoing chemotherapy, which may result from the variety of methods used. The majority of studies dealing with this topic indicate that patients with cancer diagnosis more often have depressive disorders than the general population (18). Zielińska-Więczkowska and Betłakowski demonstrated that the severity of depressive symptoms among cancer patients was higher after chemotherapy than before chemotherapy (19). In the present study, we found that the severity of depressive symptoms diminished after the completed chemotherapy cycle compared to the value before the start of treatment. This finding can be explained by the fact that the treatment completion, or even part of it, gives the patient hope for a cure. Colleoni et al (20) showed that the occurrence of depressive disorders in patients with cancer influenced the decision to start treatment and its effectiveness.

In this study, the expression of both IL-1α and IL-1β at the protein level decreased after chemotherapy. The literature indicates that IL-1β stimulates the formation of other cytokines, eg, IL-2, INF-gamma by T lymphocytes, as well as IL-6 (21). IL-1 and IL-6, among others, regulate the acute phase reaction (22). An increase in IL-1β value was observed in patients with neurodegenerative diseases, eg, Alzheimer’s disease (23,24). In addition, its increase was associated with the deterioration of age-related cognitive functions, such as reduced learning ability or memory deficits (25,26). Also Meyers et al showed in patients with acute myeloid leukemia and myelodysplastic syndrome treated with chemotherapy, significantly elevated levels of IL-1, IL-6, IL-8, TNF-alpha, compared to the control group (27).

We did not find a significant relationship between the level of interleukins and participants' cognitive efficiency. Chung et al (9) found that an increase in plasma concentration of IL-1β was associated with a decrease in the speed of response on neuropsychological tests, while an increase in IL-4 concentration was associated with an increase in response rates. Furthermore, with an increase in IL-1β and IL-6 levels, the changes in cognitive functions were more noticeable to the patients themselves (9).

Numerous studies conducted so far to evaluate cognitive functions after chemotherapy present divergent data on this subject, both confirming and denying the existence of these disorders. In addition, a significant obstacle is the lack of standard testing methods. This cognitive deficit is often referred to as mild, and attention is also drawn to its possible subjective nature, as it is often not confirmed by psychological tests. Moreover, there are reports highlighting that cognitive disorders exist before treatment (6) and therefore are not related to treatment (28). The analysis of cognitive performance in our study also provided ambiguous results. Andryszak (29) found that 43% of the studies in breast cancer patients found a lack of chemotherapy-dependent cognitive function disorders, while other studies found deficits involving attention, working and visual memory, processing ability, learning, and speech function (29). Interestingly, the overwhelming number of these studies did not confirm the relationship between depressive state and anxiety and cognitive function deterioration (29). However, Bury et al found non-specific changes in cognitive functions as well as higher severity of depressive symptoms in patients treated with chemotherapy compared with healthy participants (30), while Wefel et al demonstrated a relationship between distress and impairment of cognitive function in 35% of patients with breast cancer before supplementary treatment (31). In our study, we confirmed a decrease in cognitive performance after chemotherapy only in the Stroop test and one part of the verbal fluency test. So far, it has not been clearly defined which of the elements of cognitive functions that are reduced after chemotherapy are particularly characteristic for it. Some studies found that visual and spatial skills (32-34), working memory, depressive symptoms (33), visual memory, verbal learning ability (34), and language and memory functions (35) to be significantly affected.

Many studies have attempted to find a relationship between the level of cytokines and changes in cognitive functions as well as the severity of depressive disorders after the systemic treatment in patients suffering from cancer. Although most of them showed some deviations in cytokine concentration due to chemotherapy, the results concerning the relationship between cytokine levels and modification of cognitive functions are not clear (36,37). It seems that several methodological problems (eg, variety of research tools used, size of the studied groups) prevent the correct interpretation of the available data on chemobrain occurrence. Therefore, it is necessary to carry out further studies to assess the deficit in cognitive functions in patients with cancer in order to determine the prevalence of this phenomenon (38).

The limitations of the study are the size of the studied groups, as well as the disproportions in the demographic distribution of study participants in each of them (sex).

It is important to expand the research into the relationship between immune system components (cytokines) and severity of depression in cancer patients treated with chemotherapy. Depression is one of the most common disorders associated with cancer, with many mutual two-way mechanisms of action. Another important issue is the chemobrain phenomenon. Some studies indicate that cancer-related cognitive disorders may be long-lasting and occur even after 5 to 10 years after treatment (39). Owing to the complexity and prevalence of the chemobrain phenomenon, it is necessary to seek further relationships between cognitive function deficits and the applied chemotherapy in patients with cancer.
